# Overburden movement laws in thin bedrock workfaces under thick backfilled loose bodies

**DOI:** 10.1038/s41598-025-25889-2

**Published:** 2025-11-25

**Authors:** Wenxiang Zheng, Chao Wei, Shuai Guo, Zhipeng Zhuang, Changhui Shi

**Affiliations:** Institute of Mining and Coal, Inner Mongolia University of Science and Technology, Inner Mongolia Baotou, Baotou, 014010 China

**Keywords:** Thick backfilled loose bodies, Thin bedrock, Open-pit slope, Overburden failure, Overburden movement, Energy science and technology, Engineering, Natural hazards, Solid Earth sciences

## Abstract

A detailed investigation was conducted on the geological conditions of thin bedrock workfaces under thick backfilled loose bodies in the first panel of the 22-upper coal seam at Shigetai Coal Mine. Utilizing both physical similarity modeling and numerical simulation techniques, the study comprehensively examined the overburden movement patterns and failure characteristics under these conditions. The physical simulation results indicate that as the working face approaches the open-pit slope area, the height of overburden failure gradually increases. When the thin bedrock becomes unstable due to rotation, unconsolidated materials at the slope base flow into the goaf. Subsequently, roof-cutting pressure on the thin bedrock causes simultaneous movement of backfilled loose bodies at different heights, resulting in minor surface settlement. As mining progresses, fracturing alternates periodically between roof-cutting and relief phases, ultimately expanding the surface subsidence zone. The maximum subsidence of the thin bedrock remains less than the mining height of the coal seam. Numerical simulations reveal that when the working face leaves the open-pit slope, the overburden failure height rises rapidly. Upon entering the thin bedrock workface area, advanced fractures develop, and the thin bedrock undergoes full-thickness fracturing in small cycles with intervals of approximately 10 m. The advanced stress fluctuates around the original rock stress, and the movement of backfilled loose bodies quickly reaches the surface. When re-entering the open-pit slope, the development of advanced fractures ceases, and the overburden failure height increases again. The advanced stress gradually rises and stabilizes in the waste dump area. Subsidence in the thin bedrock workface area consistently exceeds that in the open-pit slope area. Maximum subsidence values at different measurement heights are observed within the thin bedrock zone and shift toward the slope base as height increases. When exiting the slope, subsidence in the open-pit area is greater than when entering it. This research elucidates the dynamic fracturing mechanisms and movement patterns of overburden in thin bedrock workfaces beneath thick backfilled loose bodies, providing a theoretical foundation for safe mining in similar geological settings.

## Introduction

Coal resources remain the cornerstone of China’s energy structure, sustaining their dominant position in the national energy portfolio for the foreseeable future. Thick backfilling technology effectively supports and reinforces goaf areas by injecting substantial filling materials, thereby mitigating roof collapse and restraining fracture propagation. The unconsolidated strata, comprising sand, soil, gravel, and pebble layers, exhibit significantly lower mechanical strength compared to bedrock formations. When mining-induced effects propagate to the surface through these strata, pronounced fluid-like deformation characteristics manifest^1,2^. Professor Huang Qingxiang^3–7^ established the structural theory for thin bedrock roofs and identified load-transfer mechanisms in thick sandy-clay overburden; Du Feng and Bai Haibo et al.^8–11^ defined thin bedrock as single or bi-layer composite key strata; Lian Wangqing^12^ demonstrated accelerated bedrock failure and intensified periodic weighting with decreasing bedrock thickness. Manifestations of strata pressure in thin bedrock faces correlate with both post-failure structures of thin bedrock^13–15^ and overlying clay layers^16^. Li Jianghua et al.^17^ revealed load-transfer effects in overburden during thin bedrock mining; The work by Li Xinlei et al.^18,19^ provides key methodologies for effectively controlling strata pressure behavior in deep, high-stress coal seams and for optimizing the stability of roadways driven beneath coal pillars.Zhai Xinxian et al.^20^ employed UDEC simulations to investigate bedrock deformation and strata behavior under extremely thick unconsolidated layers; Guo Longhui et al.^21^ analyzed fracture evolution in overburden during thin bedrock extraction; Wang Beifang et al.^22^ proposed instability criteria for shear-collapse structures in shallow covers with thick unconsolidated strata; Zhang Guangchao et al.^23^ studied failure patterns in weak overburden beneath mega-thick unconsolidated layers; Wang Zhenwei et al.^24^ compared overburden damage characteristics across mining methods in weathered-zone roofs under thin bedrock and thick unconsolidated strata.

While substantial research has been conducted on overburden movement and strata pressure under shallow cover, thick unconsolidated strata, and thin bedrock conditions, critical unknowns remain regarding the structural characteristics of overburden composed of thick backfilled granular materials overlying thin bedrock. Specifically, the mechanisms of overburden movement and the fracture and caving behavior of thin bedrock are not well understood—particularly since surface subsidence patterns in such geological settings differ markedly from those observed under conventional mining conditions^25,26^. This study employs theoretical analysis, physical modeling, and numerical simulation to systematically investigate the structural evolution of compound overburden (backfill-thin bedrock), overburden displacement dynamics, and fracture propagation and collapse mechanisms in thin bedrock. Using actual mine geotechnical conditions as the engineering context, the research aims to provide scientific guidance and practical experience for disaster prevention in similar mining environments.

### Engineering geological background

**Fig. 1:**
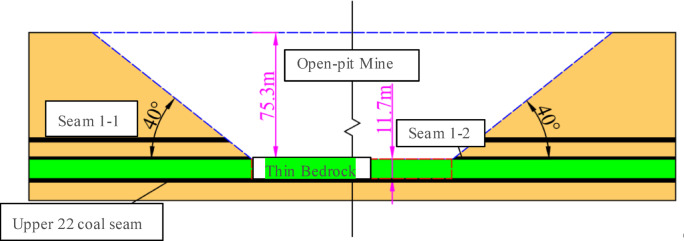
Stratigraphic diagram of 22^Upper^ seam mining area.

Located in Daliuta Town, Shenmu City, Shaanxi Province, Shigetai Coal Mine employs a combined drift-inclined shaft-vertical shaft development system across two mining levels. The 1–1 coal seam has been fully extracted, and the 1–2 seam is in its final extraction phase. Current operations focus on Seam 31 and Seam 22 (Panel 3), as well as the Upper 22 coal seam (Panel 1)—the upper split of Seam 22, which has a thickness of 1.6–2.4 m (average 2.0 m), a near-horizontal dip of 1–3°, and a stable geological structure. The mining zones include: (1) conventional mining zones (①, ⑤) overlain by surface dump sites; and (2) thick backfilled unconsolidated mass zones, subdivided into open-pit slope areas (②, ④) and thin bedrock areas (③), as detailed in Fig. 1.

## Similarity simulation research

### Instrumentation layout and excavation process in similarity simulation

Similar material simulation experiments involve constructing a scaled-down model and conducting excavation tests based on the actual geological conditions of the studied working face, following specific similarity criteria. The accuracy of these experiments depends on the precision of these criteria. However, in practice, achieving complete consistency between the similarity criteria and field conditions is often challenging. Therefore, further research is necessary to refine the determination of similarity conditions to ensure that the final model more accurately represents the actual field situation. Common similarity criteria include geometric similarity, kinematic similarity, and unit weight similarity. In this experiment, the following similarity constants were determined through calculation: Geometric similarity involves scaling down the prototype dimensions to a manageable model size, set at a ratio of 1:100. Kinematic similarity requires that velocity, acceleration, direction, motion time, and trajectory at corresponding points in the prototype and model remain consistent or follow a fixed ratio, with a similarity ratio of 10. Unit weight similarity, considering the mechanical and physical properties of the similar materials, was set at a ratio of 1.67 between the prototype and the model. Additionally, the initial states of the two systems must be similar, including structural characteristics such as rock mass structure, bedding, joint distribution, and fault distribution.

In mining physical similarity simulation experiments, aggregates such as river sand, loess, bean-sized sand, and fly ash, along with binders like gypsum and whiting, are selected. These materials must meet the following criteria: stable mechanical properties, similarity to the prototype, predictable property variations with mix proportions, ease of preparation, rapid setting, low cost, and environmental friendliness. The coal seam is simulated using a mixture containing fly ash, while the loess layer is prepared by blending soil with edible oil. The backfill material consists of river sand, bean-sized sand (5–20 mm), and loess in a ratio of 5:2:1, with water added at one-twentieth of the total mass.

**Fig. 2:**
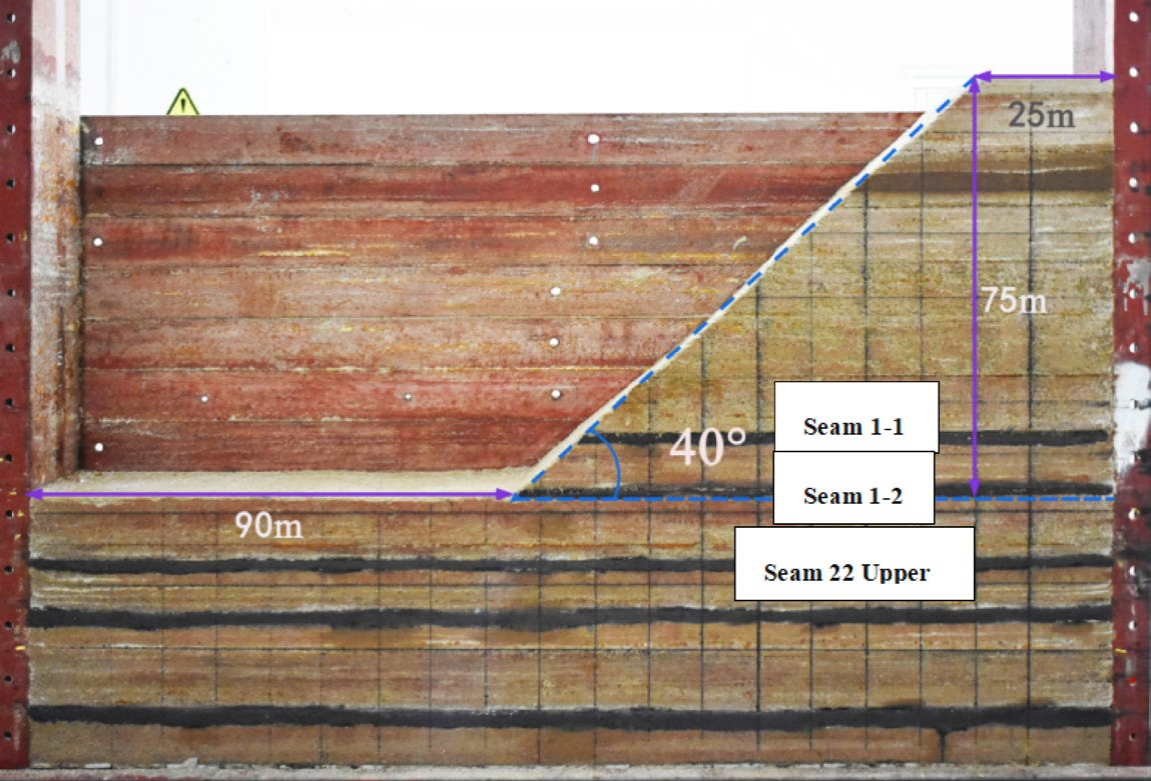
Model after the excavation of the open-pit mine.

In the physical similarity simulation experiment, aggregates and cementitious materials were first weighed according to the prescribed proportion formula, followed by initial dry mixing. A specified amount of water was then added to the mixture for further blending. The prepared material was poured onto the test platform, manually leveled, compacted using a ramming iron block, and surface-finished to ensure that each rock stratum achieved optimal flatness and horizontality. For thicker overburden strata, a layered construction method was employed, controlling each layer’s thickness to within 2 cm. Mica powder was applied between layers to serve as a parting agent, while artificial joints were prefabricated during the layering process. Based on the geological conditions of the Shigetai 22 Upper Coal First Panel, the model open-pit mine was excavated down to the floor of the 1–2 coal seam, with a slope angle set at 40 degrees. Due to the test platform’s size constraints, the simulation specifically focused on the section where the working face emerges from the open-pit slope and transitions into the thin bedrock zone. The resulting model after cutting is shown in Fig. 2.

**Fig. 3:**
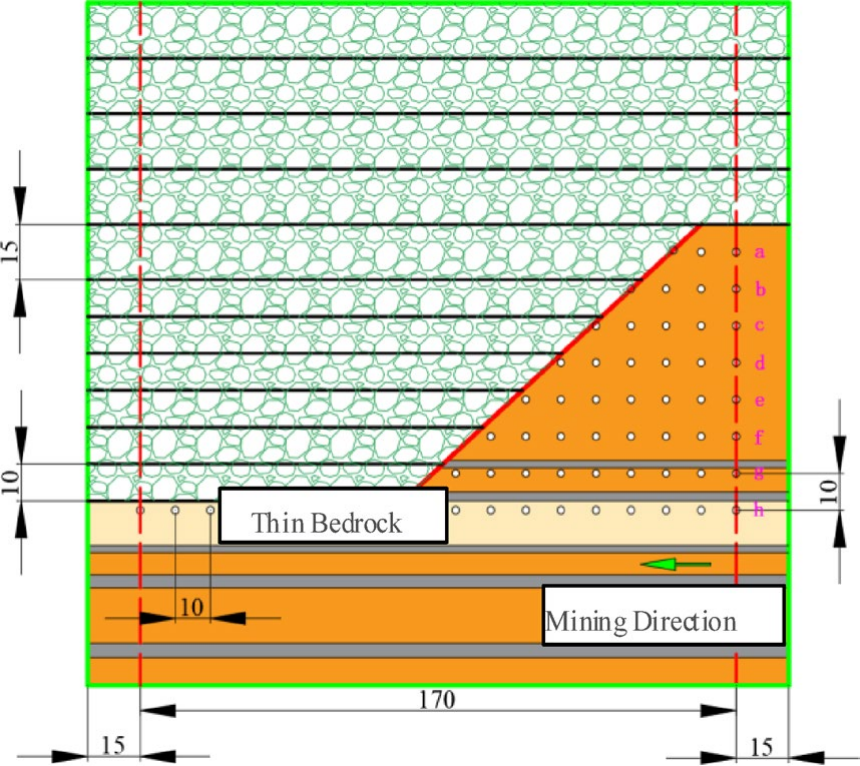
Instrumentation layout diagram.

**Fig. 4:**
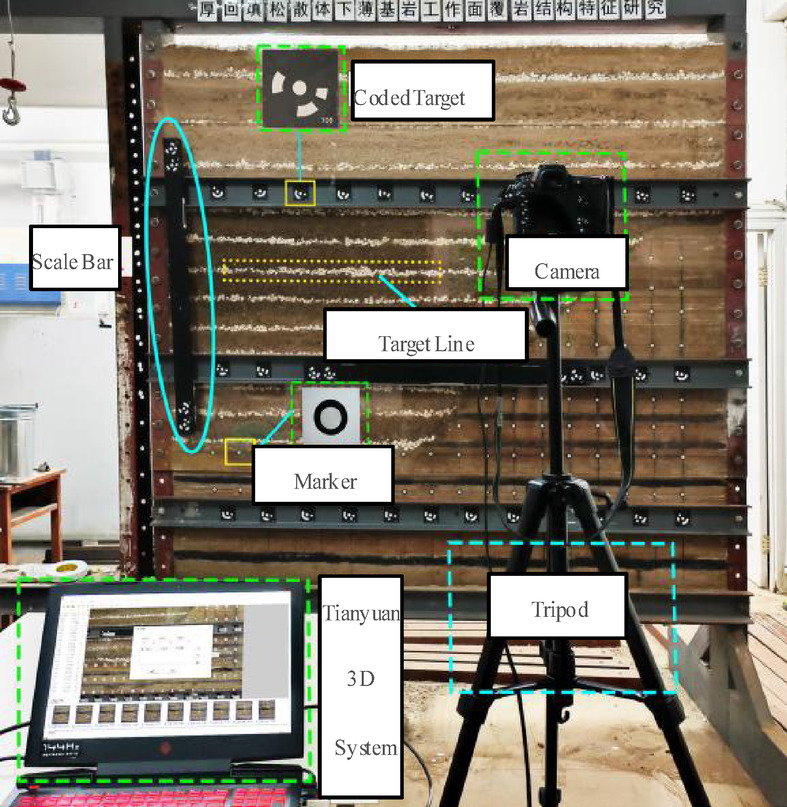
Field setup of photogrammetric system.

The similarity simulation monitored overburden failure/displacement using the Tianyuan 3D Photogrammetry System^27^. Observation points followed an “orthogonal grid pattern” with 59 retroreflective targets arranged in 8 rows (a–h, bottom-to-top) and 17 columns at 10-cm uniform spacing. Mining advanced westward (right→left), starting with a 5-cm cut on the east, progressing in 2-cm increments (Figs. 3 and 4)^28^.

The mining simulation uses a temporal similarity ratio that matches the actual advance rate at the Shigetai Coal Mine. Extraction progresses from right to left, starting with an initial 5 cm cut on the right side, followed by successive 2 cm advancement cycles until completion. The entire process is documented through photographic records^29,30^.

### Evolution mechanisms of underlying overburden fracturing beneath open-pit slopes

**Fig. 5:**
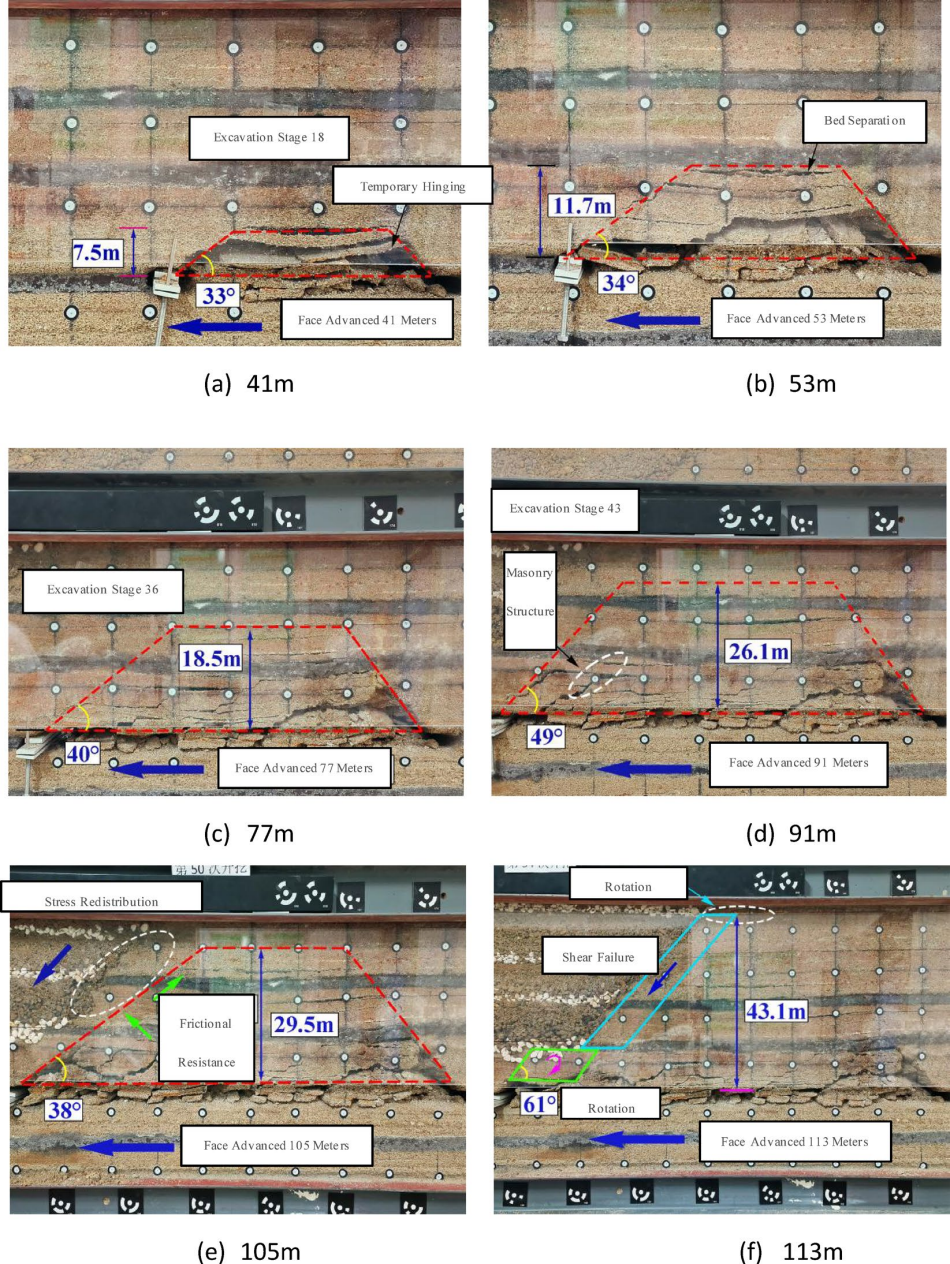
Overburden fracturing evolution beneath open-pit slopes.

Figure 5 illustrates the progressive evolution of overburden fracturing characteristics as mining advances. At 41 m of advancement, the 5-meter-thick fine-grained sandstone experienced initial caving, with significant bed separation observed in partial strata. A temporary voussoir beam structure formed on the side of the open-off cut, resulting in an overburden failure height of 7.5 m and exhibiting a trapezoidal damage pattern with a fracture angle of 33°. At 53 m, the first periodic fracturing of the main roof occurred, expanding the trapezoidal failure zone. Bed separation extended down to the floor of Seam 1–2, yielding a failure height of 11.7 m and a caving angle of 34°. By 77 m, fracture propagation reached a height of 18.5 m with a caving angle of 40°. Upon advancing to the slope toe at 91 m, the fracture height increased to 26.1 m, and the caving angle peaked at 49°. At 105 m, as the face entered the thin bedrock zone, the failure height reached 29.5 m with a caving angle of 38°. At this stage, the destruction remained confined within the slope area. The marginal uncaved zone was temporarily stabilized due to support from the trapezoidal failure area and frictional resistance from the bedrock. Additionally, the overburden structure at the slope toe transitioned to thin bedrock overlain by thick granular material. The granular material slid along the slope, creating a low-stress zone at the toe that inhibited further overburden failure, thereby temporarily stabilizing the slope margin. Upon reaching 113 m, the thin bedrock underwent rotational instability and initial fracturing, increasing the sliding space at the margin and disrupting equilibrium. This triggered overall shear failure, culminating in a maximum failure height of 43.1 m.

Overall, as the longwall face advanced beyond the open-pit slope, the overburden caving exhibited a trapezoidal pattern that continuously expanded with mining, following the main roof fracture. When the face entered the thin bedrock zone, the bedrock failure within the open-pit slope area had not yet stabilized.

### Overburden fracturing mechanisms under thick backfill overlying thin bedrock

**Fig. 6:**
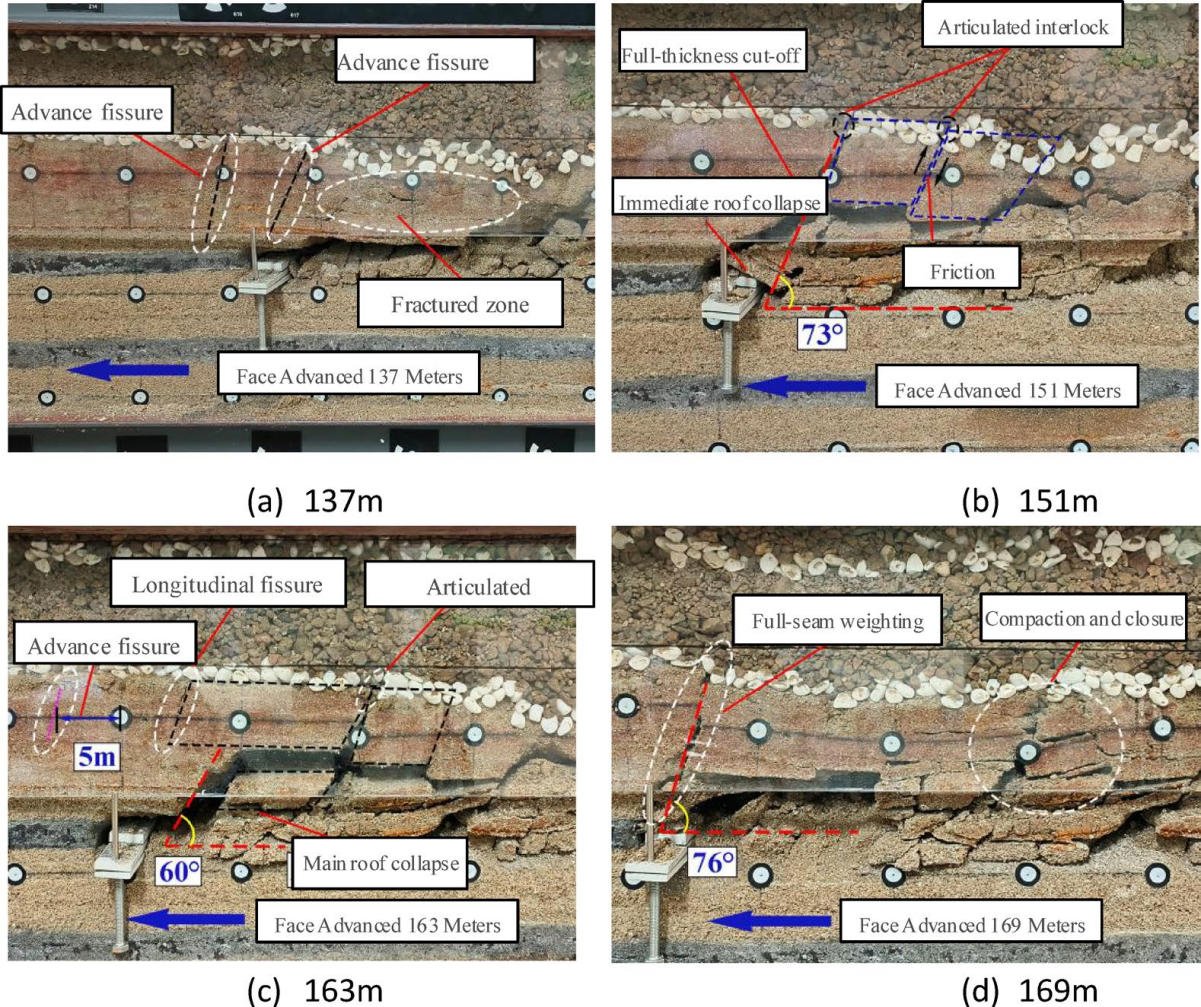
Overburden Failure Under Thick Backfill-Thin Bedrock.

Figure 6 illustrates the evolutionary sequence of overburden fracturing under thin bedrock conditions during longwall mining. At 137 m of advancement, vertical fractures appeared in the overlying strata, with advanced fractures located 5 m ahead of the coal face. The roof in the goaf fractured and collapsed. Upon reaching 151 m, the first roof cutting and weighting event occurred, involving full-thickness caving of the thin bedrock at a caving angle of 73°. A temporary voussoir beam structure began to form above the main roof. By 163 m, advanced fractures continued to develop 5 m ahead of the coal face. Roof rotation was temporarily restrained by the voussoir beam structure; however, the immediate roof and main roof successively collapsed, resulting in a caving angle of 60°. Toward the final mining stage at 169 m, the roof experienced another full-thickness cut along the fractures from the previous cycle, yielding a caving angle of 76°. The voussoir beam structure in the rear goaf had compacted and closed.

Overall, after the initial fracture of the thin bedrock, advanced fractures began to develop. Subsequently, the failure of the thin bedrock exhibited a layered fracturing pattern. When the face advanced to 151 m, the thin bedrock experienced full-thickness fracturing. Thereafter, the failure mechanism alternated between full-thickness fracturing and layered fracturing.

### Migration behavior of thick granular backfill

**Fig. 7:**
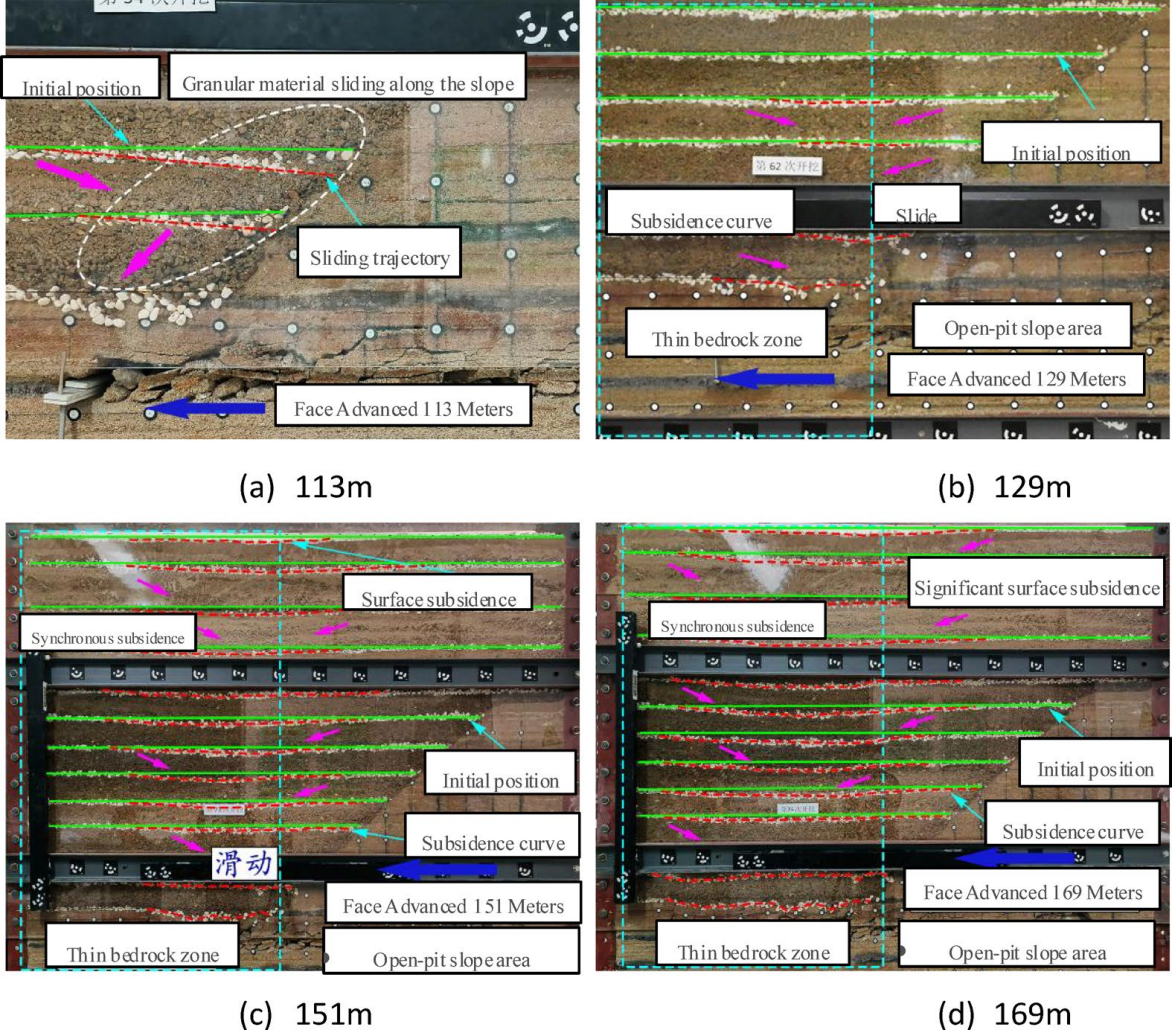
Migration of thick granular backfill.

Figure 7 illustrates the migration and evolution of thick granular backfill. Advancing to 113 m induced rotational subsidence of the bedrock at the slope toe, triggering slight granular sliding within a 20-meter-high zone toward the subsiding area. Progressing to 129 m exacerbated bedrock fracturing, driving the overlying granular flow toward the goaf from both the slope and the working face. At 151 m, the first roof-cutting pressure expanded the bedrock failure zone—compacted under the overburden load—while accelerating migration with initial surface subsidence and progressive upward expansion of sliding zones. The final advancement to 169 m (second roof-cutting pressure) intensified granular subsidence, causing significant surface settlement with extended coverage. Granular survey lines at varying depths exhibited synchronized subsidence with minimal differential displacement (< 5% variance), culminating in pervasive deformation.

Overall, the initial fracture of the thin bedrock triggered the first migration of granular material. Subsequently, the extent of granular material migration continued to expand. When the first full-thickness fracture of the thin bedrock occurred, the influence of granular migration reached the ground surface. The granular material lacks any internal self-stabilizing structure and exhibits fluid-like mobility. The variation in subsidence observed across measurement lines at different burial depths is attributed to the sliding of granular material both ahead of and behind the goaf toward the mined-out area, with frictional resistance between granular particles also contributing.

### Evolution law of overburden fracture angle

**Fig. 8:**
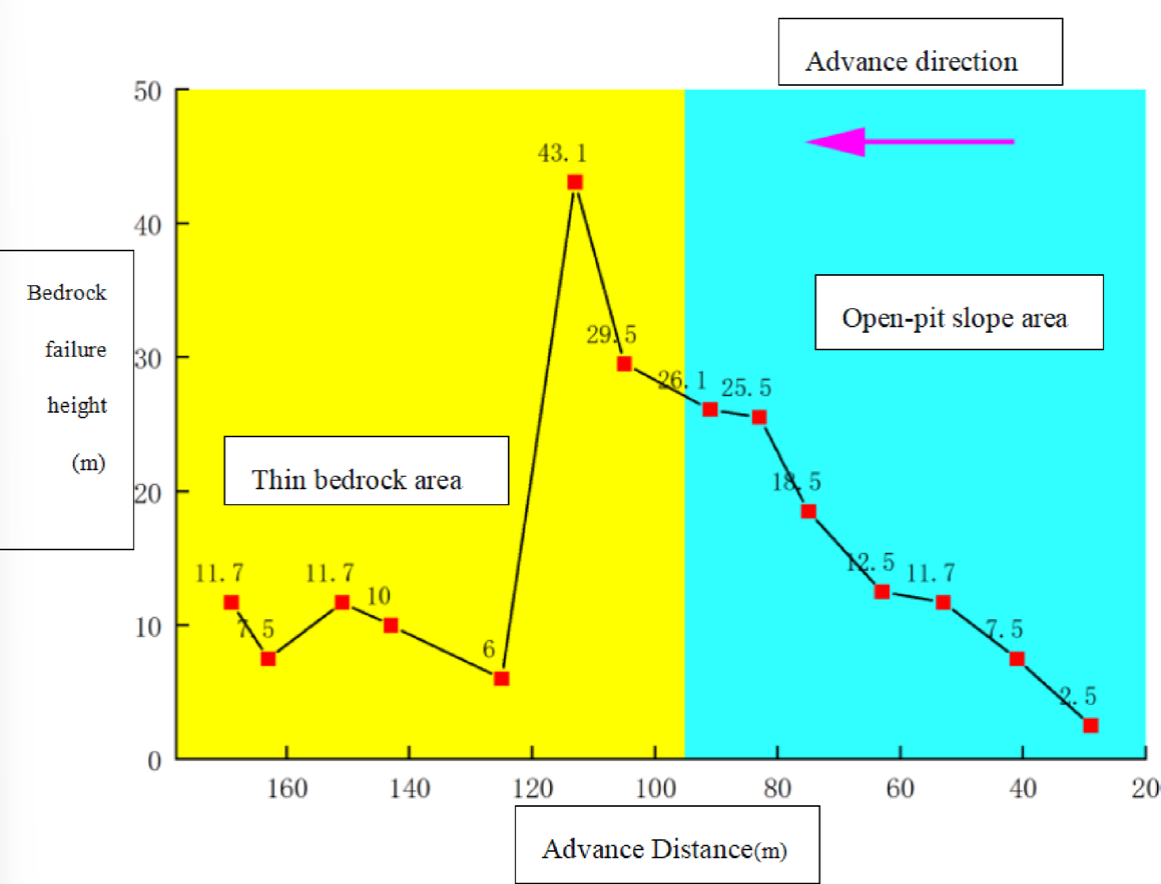
Evolution curve of bedrock failure height.

Figure 8 illustrates the evolutionary pattern of the overburden fracture angle as the working face advances. Beginning at the open-off cut position, the overlying strata of the coal seam gradually collapse. When the working face reaches 41 m, the main roof experiences its first weighting, and the overburden exhibits trapezoidal failure with a fracture angle of 33°. At 75 m, the fracture angle increases slightly to 36°. During the 75–113 m stage, the overburden structure undergoes significant changes, with the fracture angle rapidly rising from 36° to 49°, representing a 36% increase. At 105 m, the working face enters a thin bedrock area, causing the fracture angle to briefly drop to 45°, primarily due to the intact slope toe. By 113 m, rotational instability occurs at the slope toe, causing the fracture angle to surge to 61°. Subsequently, the thin bedrock forms a cantilever beam structure with developed pre-cracking, and the fracture angle fluctuates between 50° and 60°. At 151 m, the first full-thickness cutting occurs, with the fracture angle reaching 73°. By 163 m, periodic fracturing of the main roof causes the fracture angle to decrease to 57°. At 169 m, the second full-thickness cutting takes place, and the fracture angle reaches its maximum value of 76°, after which the changes stabilize.

Overall, as the working face advances and the overburden structure evolves, the fracture angle undergoes three stages: slow growth, rapid increase, and eventual stabilization, reflecting the progressive failure dynamic process from the slope area to the thin bedrock area.

## Numerical simulation study

### Numerical simulation scheme

UDEC is a two-dimensional numerical analysis software based on the discrete element method, designed to study the mechanical behavior of discontinuous media and complex discontinuous geometric features. It offers high computational efficiency and excellent stability. Therefore, UDEC was chosen to simulate overburden movement patterns in a working face with thin bedrock beneath thick backfilled loose bodies.

A simplified numerical model was developed using the UDEC^31^ code, measuring 400 m in length and 160 m in height, based on the geological conditions of the Shigetai Coal Mine. The model was divided into three zones from left to right: the thin bedrock zone (x = 0 to 190 m), the open-pit slope zone (190 to 280 m), and the waste dump zone (280 to 400 m). The bedrock was simulated using a brick model, while the waste dump material was represented by a Voronoi tessellation model with polygon edges measuring 3 m in length. This modeling approach effectively represents the cemented structures and failure processes in natural rock masses, making it suitable for analyzing overburden movement beneath thick backfilled loose bodies.

The model features a free upper boundary, with fixed constraints applied to the left, right, and bottom boundaries. An 80-meter-wide boundary zone was incorporated on each side to minimize boundary effects. The rock mass joints were simulated using the Coulomb slip model, while both the intact blocks and the Voronoi^32^ assembly were governed by the Mohr-Coulomb constitutive criterion. The physico-mechanical parameters of the rock materials are listed in Table 1, and the mechanical parameters of the joints are provided in Table 2.

**Table 1:** physico-mechanical parameters for the rock materials.

Material type	Density (kg/m³)	Bulk modulus (GPa)	Shear modulus (GPa)	Cohesion (MPa)	Friction angle (°)	Tensile strength (MPa)
Backfill material	2000	0.50	0.10	0.50	6	0.04
Aeolian sand	1800	2.70	1.50	0.70	12	0.06
Loess	2000	5.00	1.00	0.80	8	0.04
Coal	1460	1.50	1.40	1.20	22	0.98
Sandy mudstone	2500	8.30	4.60	0.90	24	1.20
Siltstone	2450	7.30	3.80	1.50	28	1.27
Fine sandstone	2250	7.60	5.10	1.60	32	2.10
Medium sandstone	2400	10.0	7.30	2.30	35	1.60

**Table 2:** Mechanical parameters for the joints.

Material type	Normal stiffness (GPa)	Shear stiffness (GPa)	Cohesion (MPa)	Friction angle (°)	Tensile strength (MPa)
Backfill material	0.1	0.08	0.01	6	0.01
Aeolian sand	0.4	0.3	0.2	8	0.01
Loess	0.6	0.4	0.2	10	0.05
Coal	0.8	0.5	0.5	16	0.7
Sandy mudstone	2.6	1.4	0.8	18	0.9
Siltstone	4.0	3.0	1.3	20	0.8
Fine sandstone	3.2	1.8	1.1	19	1.0
Medium sandstone	2.8	1.4	1.5	22	1.1

**Fig. 9:**
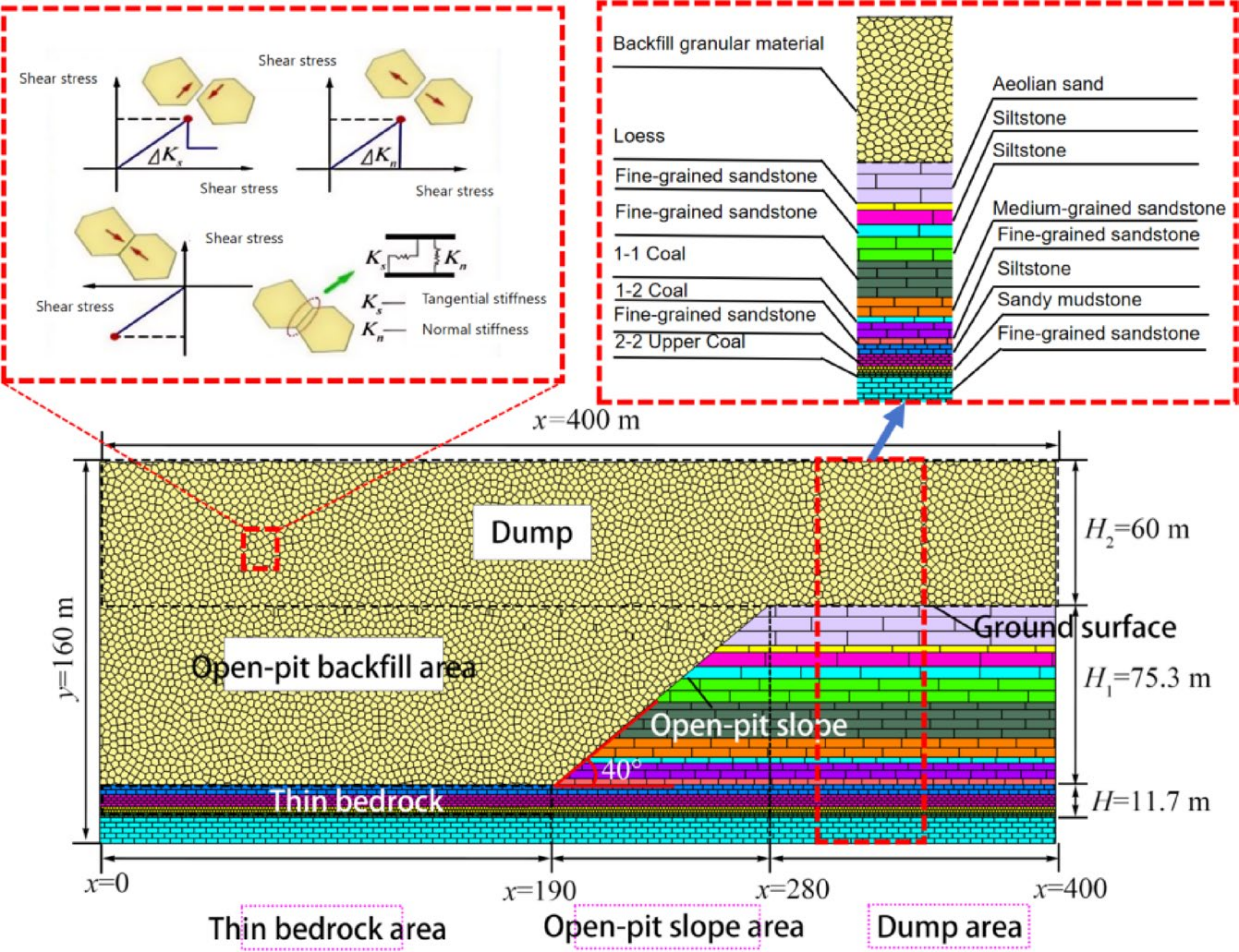
Initial numerical model configuration.

This study investigates the overburden movement patterns in a working face characterized by thin bedrock underlying thick backfilled loose materials. It simulates the failure characteristics of the overlying strata as the face advances into, through, and beyond the open-pit slope and thin bedrock zones. The simulation procedure includes the following steps: model establishment, initial stress equilibrium, panel extraction (with each excavation step advancing 10 m, totaling 240 m), and calculation to equilibrium until mining completion. The initial model is shown in Fig. 9.

### Overburden failure characteristics upon exiting open-pit slope

**Fig. 10:**
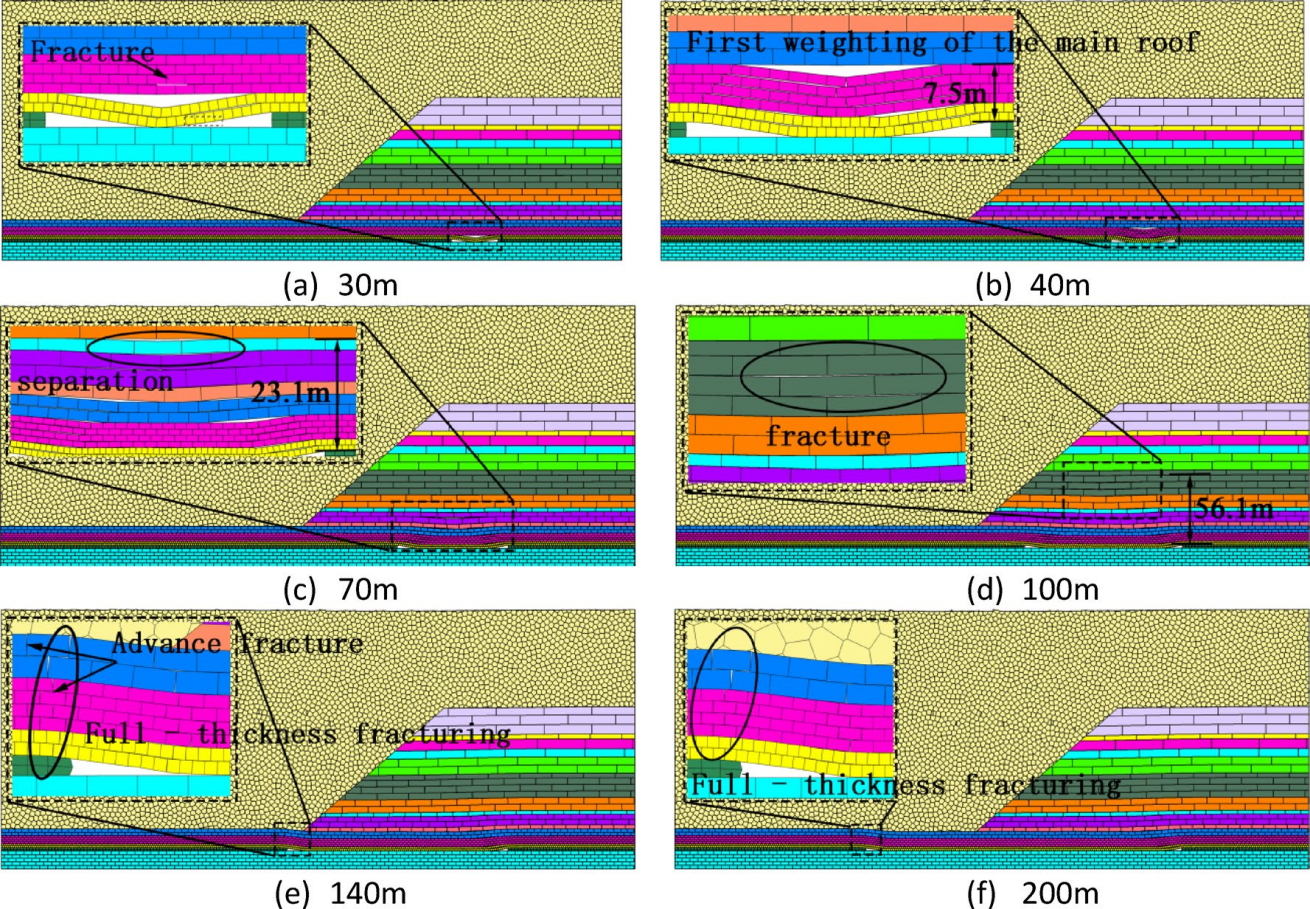
Overburden failure characteristics upon exiting open-pit slope.

Figure 10 illustrates the evolution of overburden failure characteristics as the working face exits the open-pit slope. At an excavation distance of 30 m, the immediate roof composed of sandy mudstone with low tensile strength collapsed, forming a caving zone of 2.5 m in height and initiating micro-fractures in the main roof. By 40 m, the 5 m-thick fine-grained sandstone reached its critical span and fractured completely, elevating the caving height to 7.5 m. As the face advanced to 70 m, the overlying siltstone and strata up to Seam 1–1 fractured, leading to bed separation beneath the coal roof and increasing the failure height to 23.1 m. At 100 m, rapid fracture propagation through the fine sandstone surged the failure height to 56.1 m, after which fracture development stabilized within the slope domain. Upon entering the thin bedrock zone at 140 m, full-thickness bedrock fracturing occurred immediately, while the granular backfill remained largely intact. Finally, at 200 m, periodic fracturing of the bedrock continued; however, the fluidity of the granular material suppressed the development of internal fractures, causing the overburden movement to transition into a stabilized cyclic pattern.

### Overburden failure characteristics upon entering open-pit slope.

**Fig. 11:**
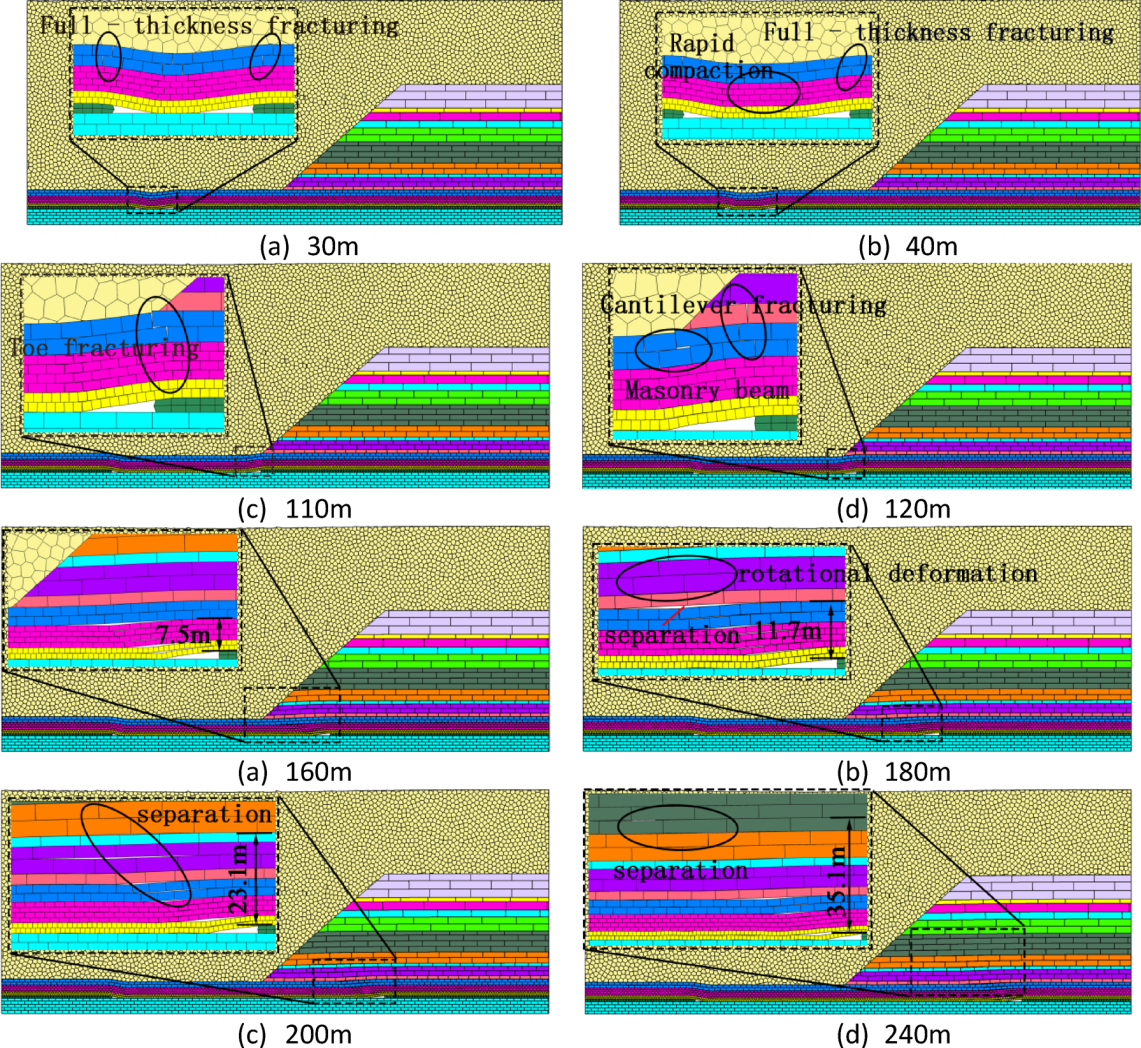
Overburden failure characteristics upon entering open-pit slope.

Figure 11 illustrates the evolution characteristics of overlying strata failure as the working face advances toward the open-pit slope. When the face advanced to 30 m, the thin bedrock experienced full-thickness fracturing, and the overlying strata fracture line extended ahead of the coal wall. At 40 m, the first periodic weighting occurred, with the thin bedrock fracturing fully again and the bedrock in the middle of the goaf gradually compacting. Thereafter, the thin bedrock exhibited a regular pattern of full-thickness fracturing. By the time the face advanced to 110 m, the thin bedrock underwent full-thickness fracturing at the slope toe, while longitudinal fractures continued to develop ahead of the coal wall. At 120 m, the working face officially entered the open-pit slope area, and the height of overlying strata failure extended upward. The overlying 1–2 coal seam and fine-grained sandstone above the thin bedrock at the slope fractured, while the siltstone within the thin bedrock formed a “voussoir beam” structure behind the working face. At 160 m, separation and fracturing occurred between the immediate roof and the main roof, with only slight bending observed in the strata above the main roof. The height of overlying strata failure was 7.5 m. At 180 m, the separation further developed into the siltstone above the immediate roof, and the failure height increased to 11.7 m. By 200 m, the failure height reached 23.1 m, and by 240 m, it further extended to 35.1 m.

### Overburden movement behavior upon exiting open-pit slope

**Fig. 12:**
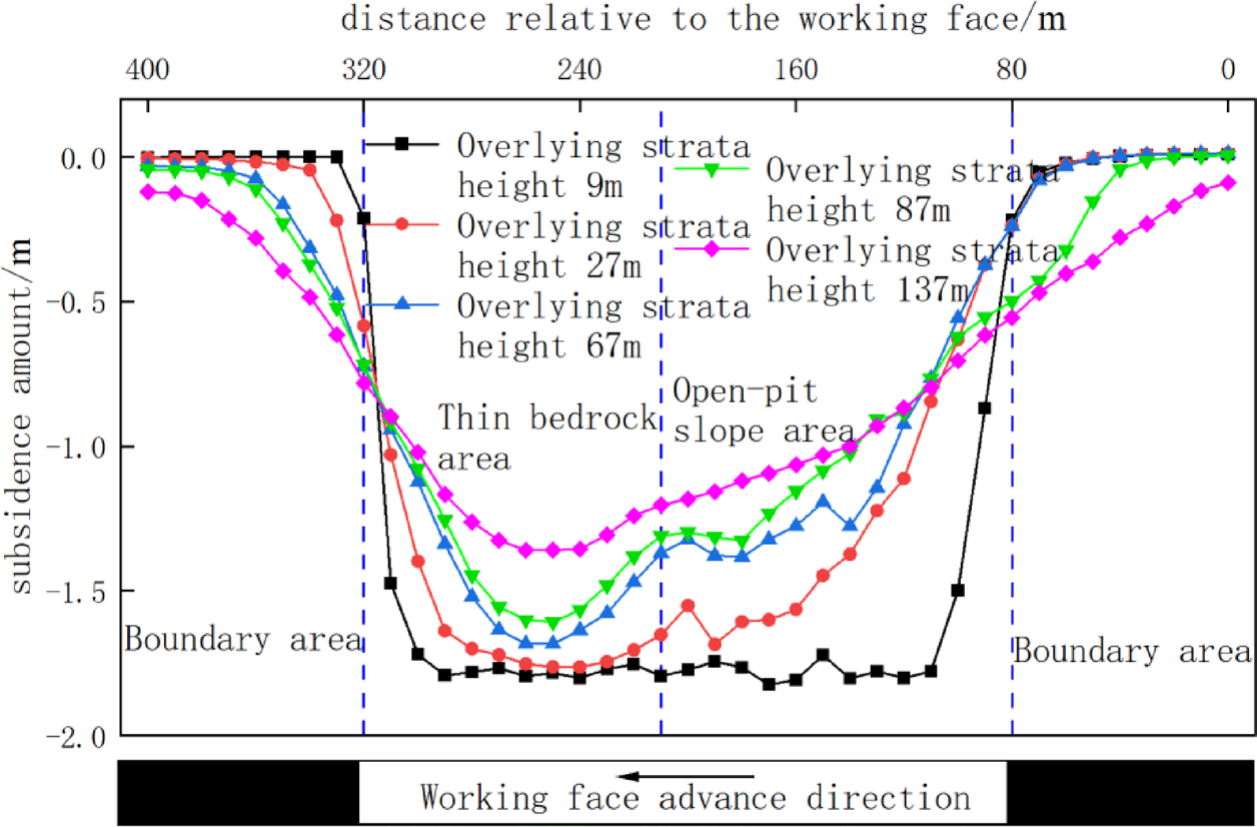
Overburden subsidence vs. height upon slope exit.

Figure 12 quantifies the final subsidence of monitoring lines at varying overburden heights upon exiting the open-pit slope. At 9 m height (within thin bedrock caving zone), all lines exhibited significant subsidence (~ 1.8 m), less than mining height. At 27 m height, lines in thin bedrock backfill showed an inverted-trapezoid subsidence curve, with maximum subsidence (1.76 m) in thin bedrock; slope zone subsidence decreased sharply from crest to toe. At 67 m height, backfill in thin bedrock formed a V-shaped curve peaking at 1.68 m centrally. Here, slope backfill displacement slightly decreased near the toe due to lateral compression from bedrock subsidence, while slope bedrock had compacted to stable subsidence. At 87 m height, subsidence trends resembled the 67 m case (max: 1.60 m, merely 4.7% reduction). At 137 m height (near surface in waste dump), a hooked-shaped curve emerged with moderated slope deformation but pronounced backfill subsidence in bedrock zones.

### Overburden movement behavior upon entering open-pit slope

**Fig. 13:**
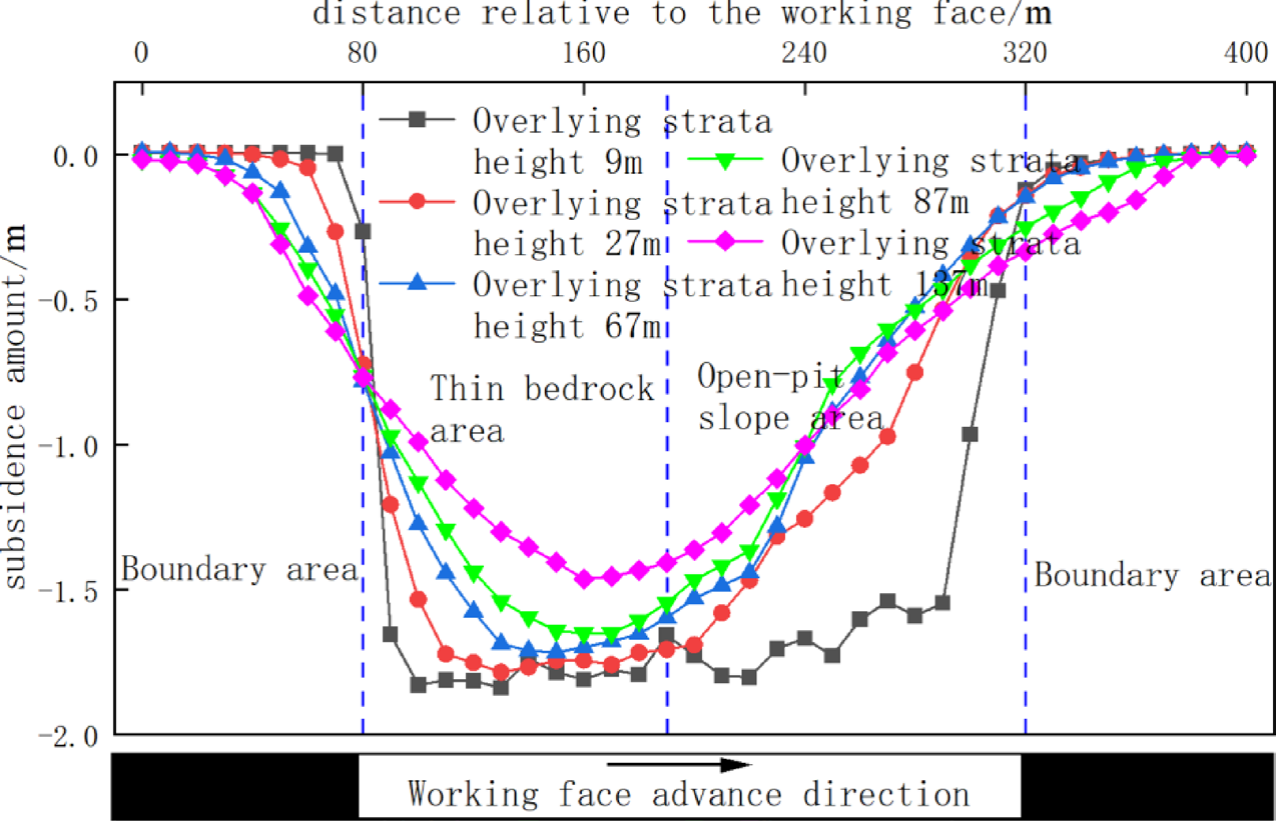
Subsidence vs. height upon slope entry.

Figure 13 quantifies final subsidence of monitoring lines at varying overburden heights upon entering the open-pit slope. At 9 m height, significant subsidence occurred with larger magnitudes in thin bedrock zones than slope zones, exhibiting an inverted-trapezoid curve. At 27 m height, bedrock subsidence in thin bedrock zones remained comparable to the 9 m case, while slope zone subsidence gradually decreased from crest to toe, maintaining an inverted-trapezoid profile. Advancing to 67 m height transformed the curve into a V-shape peaking at 1.72 m centrally in thin bedrock. At 87 m height, subsidence trends slightly decreased (max: 1.65 m) but mirrored the 67 m pattern. Finally, at 137 m height, maximum subsidence reduced to 1.46 m and shifted toward the slope toe, with thin bedrock and slope zones converging toward symmetrical distribution though mean subsidence in bedrock still exceeded slope areas.

This chapter’s numerical simulation comparative study reveals that when the working face exits the open-pit slope, its front abutment pressure follows an evolution pattern of “stabilization → rapid increase → stabilization. when entering the open-pit slope, it exhibits a distinct pattern of “rapid increase → rapid decrease → stabilization.” A common feature in both scenarios is that the overburden and surface subsidence in the thin bedrock area are consistently and significantly greater than those in the open-pit slope area. The movement curve displays a single-curve shape on the slope side and a funnel shape on the thin bedrock side. Comprehensive analysis indicates that the overburden subsidence induced in the open-pit slope area when the working face exits the slope is greater than when it enters. This difference has important implications for roof disaster prevention and control.

## Roof disaster prevention and control technology in working faces with thin bedrock under thick backfilled loose bodies

During mining operations in thin bedrock working faces under thick backfilled loose bodies, the limited bedrock thickness, low bedrock-to-overburden ratio, substantial thickness of unconsolidated layers, and absence of stable load-bearing structures readily induce roof-cutting ground pressure phenomena and shield support crushing accidents. Simultaneously, the overlying unconsolidated strata create conditions conducive to water seepage and accumulation. The fracturing of thin bedrock forms interconnected fissure channels, which can easily trigger water and sediment inrush disasters. Consequently, preventing roof collapse and controlling water and sediment inrushes are critical for ensuring mining safety.

### Prevention of roof cutting-type weighting

To effectively prevent roof cutting-type weighting disasters in these working faces, a comprehensive engineering and technical system must be established. First, hydraulic supports with higher load-bearing capacity should be installed to ensure adequate support strength. Simultaneously, the face advance rate should be optimized to leverage the rate effect of the mining-induced stress field and enhance the roof’s self-supporting ability. This approach should be complemented by implementing an accurate ground pressure monitoring and forecasting system, reinforcing the roadway support structure, and strictly adhering to refined operational procedures such as setting-to-the-roof moving and positive pressure moving. Together, these measures form a comprehensive prevention plan that integrates equipment reliability, process optimization, and meticulous management.

### Prevention of water and sand inrush

During mining, voids exist between the backfilled unconsolidated materials, which readily collect surface water and old goaf water from overlying workings. According to the physical similarity simulation conducted in this study, roof-cutting-type weighting phenomena are highly likely to occur in areas with thin bedrock, connecting the working face goaf with the thick backfilled unconsolidated layer and forming channels for water and sand inrush. In thin bedrock working faces, the formation of roof fracture channels is a prerequisite for water and sand inrush, with water being the key factor triggering the disaster. Therefore, before mining, geophysical methods such as electromagnetic surveys must be employed to identify water-bearing areas. These should be combined with existing hydrogeological data to implement targeted water drainage, reducing the risk of water accumulation and effectively preventing water and sand inrush disasters.

Grouting reinforcement can bind the loose backfill above the thin bedrock into a cohesive mass, effectively increasing the thickness and strength of the immediate roof layer. This process prevents large-scale roof fracturing, reduces the formation of water and sand inrush channels, and ultimately significantly lowers the risk of disasters.

### Prevention of water and sand inrush

#### Overburden failure at various base-dispersion ratios

**Fig. 14:**
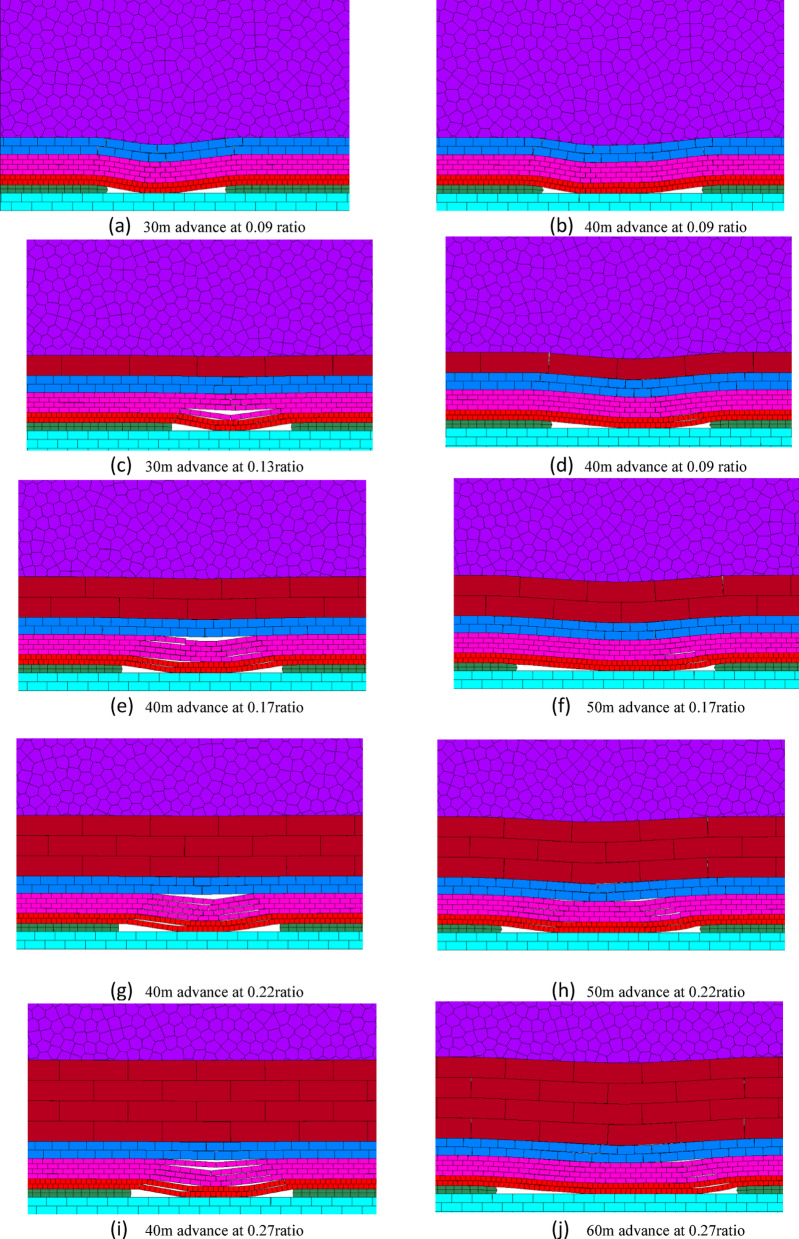

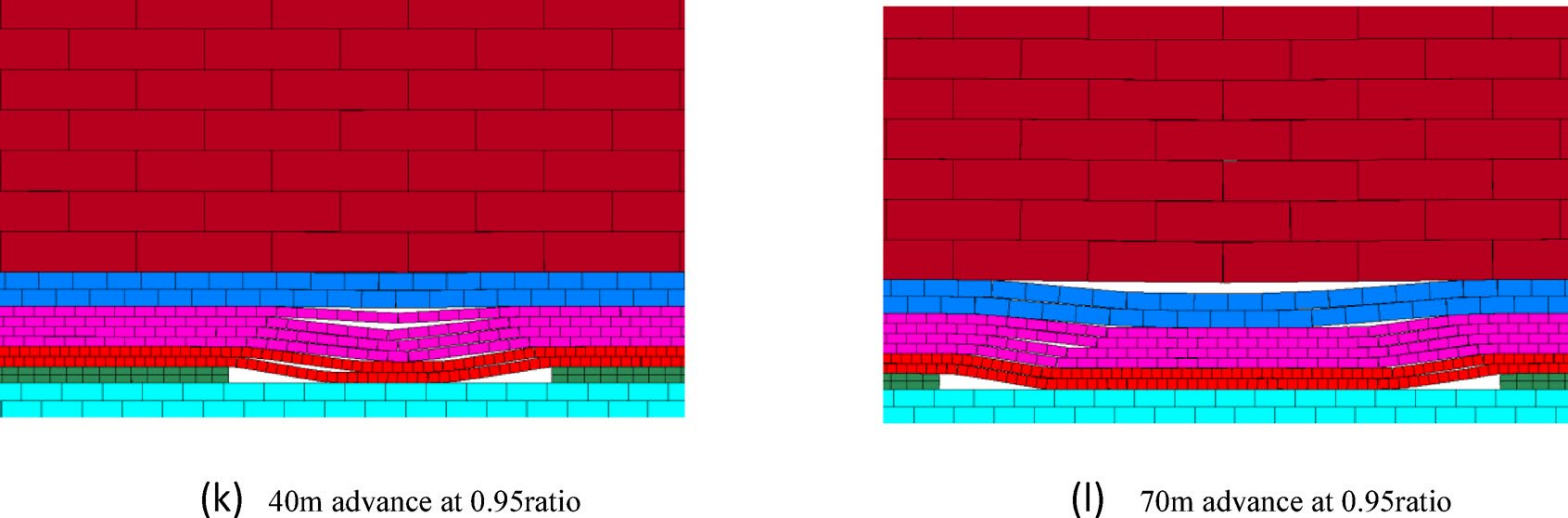
Overburden failure vs. waste-to-bedrock ratio.

Figure 14 illustrates the overburden failure patterns during the early mining stage under different waste-to-bedrock ratios. As the ratio increases from 0.09 to 0.95, the failure characteristics evolve significantly: at low ratios (0.09–0.17), the thin bedrock undergoes immediate and full-thickness fracturing following the advance of the working face, accompanied by extensive longitudinal cracks. At moderate ratios (0.22–0.27), a transitional behavior emerges—the bedrock no longer fails as a monolithic layer, unconsolidated zones may form in the middle of the goaf, and a self-stable voussoir beam structure begins to develop, with reduced crack propagation. When the ratio reaches a high value (0.95), only minor transverse cracks appear in the overlying bedrock, and its collapse is significantly delayed, indicating that the load from the waste material has minimal influence on bedrock stability.

Comprehensive analysis indicates that the waste-to-bedrock ratio plays a decisive role in the overburden failure mode and the load-bearing capacity of the bedrock. When the ratio is below 0.27, the first weighting of the working face triggers overall full-thickness fracture or chain instability in the overlying bedrock, accompanied by through-going longitudinal cracks, resulting in relatively low load-bearing capacity of the bedrock. Once the ratio exceeds 0.27, the behavior of the overburden undergoes a fundamental change: the bedrock no longer experiences full-thickness fracture, longitudinal cracks cease to propagate through, its load-bearing capacity improves significantly, and the caving interval increases. This effectively suppresses overburden failure and greatly facilitates the maintenance of the working face roof.

## Conclusions

Focusing on the atypical geological configuration of Panel 22 − 1 in Shigetai Upper Coal Seam—characterized by 11.7 m-thin bedrock overlain by thick granular backfill—this study employs field investigations, physical simulations, and numerical modeling to investigate overburden fracturing characteristics and migration mechanisms during longwall mining. Key conclusions are summarized below:

1. Physical similarity simulations demonstrate that following initial roof weighting, overburden caving exhibits a step-shaped profile progressively expanding with mining advance; upon entering thin bedrock zones, failure propagation remains confined within open-pit slope domains. During early mining stages in thin bedrock, advanced fractures emerge ahead of the working face, with bedrock exhibiting layered fracturing until 151 m advancement triggers full-thickness shear failure. Subsequent fracturing transitions to roof-cutting mitigated periodic patterns. Notably, initial entry into thin bedrock induces rotational instability, initiating granular material movement, with cyclic fracturing governing later stages.
2. UDEC numerical modeling reveals overburden failure characteristics and granular migration patterns during face advance exiting/entering open-pit slopes and traversing thin bedrock. Upon slope exit, failure height increases rapidly after the main roof’s initial fracture. When entering thin bedrock, advanced fractures emerge internally, triggering periodic full-thickness fracturing with short spans and rapid goaf compaction. Slope entry halts fracture development, causing slight initial failure height increase followed by redevelopment. Crucially, subsidence in thin bedrock zones consistently exceeds slope areas across all phases; maximum subsidence values always occur within bedrock, shifting toward the slope toe with increasing overburden height, while slope-zone subsidence remains relatively higher during exit than entry.)
3. Based on physical and numerical simulation analyses, it has been concluded that the primary hazards in thin bedrock working faces under thick backfilled loose bodies are roof-cutting with support crushing and water-sand inrushes. Building on these findings, comprehensive measures including increased support resistance, accelerated advance rate, and supplementary technical approaches have been proposed to prevent roof collapse in thin bedrock working faces, thereby enhancing roof stability. Guided by these conclusions, engineering schemes for ground water drainage and grouting operations have been systematically designed for the working face.

## Data Availability

The research data are provided as part of this paper.

## References

[1] Huang, P., Spearing, A. J. S., Feng, J., Jessu, K. V. & Guo, S. Effects of solid backfilling on overburden strata movement in shallow depth Longwall coal mines in West China. *J. Geophys. Eng.* **15**, 2194–2208 (2018).

[2] Hu, S. P. & Yu, T. B. Study on overlying rock movement and mine pressure behavior in shallow-buried close coal multi-seam mining. *Alexandria Eng. J.* **105**, 578–587 (2024).

[3] Huang, Q. X. Study on load transfer of sand layer in main roof first weighting in shallow seam. *Rock. Soil. Mech.* **26**, 881–884 (2005).

[4] Yu, Z. B., Zhu, S. Y., Wu, Y. & Yu, H. T. Study on the structural characteristics of the overburden under Thick loose layer and thin-bed rock for safety of mining coal seam. *Environ. Earth Sci.* **79**, 9 (2020).

[5] Yang, W. F., Huang, Y. S., Meng, H. L. & Xia, X. H. Law of overburden and surface deformation in roof cutting and backfilling mining under thin bedrock in shallow buried coal seam. *Quart J. Eng. Geol. Hydroge.* **58** (2025).

[6] Zhang, Q. L. et al. Simulation and on-site detection of the failure characteristics of overlying strata under the mining disturbance of coal seams with thin bedrock and Thick alluvium. *Sensors.* **24** (2024). 10.3390/s24061748 PMC10974490 38544011

[7] Zhang, J. H. et al. Roof movement and instability fracture characteristics in shallow-buried thin coal seam conventional mining faces. *Geomech. Geophys. Geo-energ Geo-resour*. **10**, 27 (2024).

[8] Du, F. & Bai, H. B. Study on overlying strata fracture mechanism in fully mechanized top coal caving mining with Thick alluvium and thin bedrock. *J. China Coal Soc.* **37**, 1105–1110 (2012).

[9] ZHANG, J., HE, Y., YANG, T., BAI, W. & GAO, S. YAN Y. Study on the Co-Evolution mechanism of key strata and mining fissure in shallow coal seam mining. *Appl. Sciences-Basel*. **13**, 8036 (2023).

[10] Han, H. K. et al. Mining stress formation and distribution: predictive model based on overburden key strata structure. *Energy Sci. Eng.* **12**, 1551–1568 (2024).

[11] Wu, S. X. et al. Research on the mechanical characteristics of Thick alluvium on the surface subsidence features of thin bedrock deposits at depth. *Min. Metall. Explor.* **41**, 1281–1298 (2024).

[12] Lian, Q. W. & Song, X. M. Study on influence of thin bedrock thickness variation on mining pressure manifestation in Stope. *Coal Eng.* **9**, 48–50 (2012).

[13] Zhu, Z. J., Wang, P., Chen, K., Lv, F. & Hong Y.Evolution of the overlying strata structure and characteristics of ground pressure behavior under the influence of tectonic stress. *Front. Earth Sci.* **12**, 1501631 (2024).

[14] Zhang, S. K., Wang, Y. S. & Li, G. Manifestation law of mining pressure in coal seam with Thick alluvium and thin bedrock. *J. Ground Press. Strata Control*. **3**, 6–9 (1998).

[15] Fu, P., Luo, J., Yan, S. & Mu, J. L. The evolution law of mining stress concentration effect and mining pressure manifestation mechanism under different pushing methods in Valley landforms. *Sci. Rep.* **15**, 21113 (2025). 40596156 10.1038/s41598-025-06907-9 PMC12216713

[16] Fan, H. et al. Height of water-conducting fractured zone in a coal seam overlain by thin bedrock and Thick clay layer: a case study from the Sanyuan coal mine in North China. *Environ. Earth Sci.* **79**, 125 (2020).

[17] Li, J. H., Xu, Y. C., Jiang, P., Lian, Y. G. & Mou, Y. Study on load transfer characteristics of overlying strata in working face with super-thick alluvium and thin bedrock. *Coal Sci. Technol.* **45**, 95–100 (2017).

[18] Li, X. L., Ren, Z. Y., Cao, Z. Z. & Hao, R. Study on coal drawing parameters of deeply buried hard coal seams based on PFC. *Sci. Rep.* **15**, 21934 (2025). 40596559 10.1038/s41598-025-08154-4 PMC12216397

[19] Li, X. L. et al. Determination method of rational position for working face entries in coordinated mining of section coal pillars and lower sub-layer. *Sci. Rep.* **15**, 29440 (2025). 40790342 10.1038/s41598-025-15115-4 PMC12339699

[20] Zhai, X. X., Lyu, C., Tang, S. J. & Yu, C. S. Study on thin bedrock deformation and mining pressure manifestation in coal mining under super-thick alluvium. *Gongkuang Zidonghua*. **44**, 71–75 (2018).

[21] Guo, L. H., Cheng, H., Peng, S. L. & Fu, B. J. Study on evolution of separation fractures and fractal characteristics of subsidence in overlying strata during mining with Thick alluvium and thin bedrock. *Saf. Coal Mines*. **51**, 59–64 (2020).

[22] Wang, B. F. et al. Structural analysis and application of overlying rock cutting body in fully-mechanized mining face with shallow buried Thick alluvium and thin bedrock. *Rock. Soil. Mech.* **44**, 3011–3021 (2023).

[23] Zhang, G. C. et al. Failure law of weak overlying strata under super-thick alluvium. *J. China Coal Soc.* **47**, 3998–4010 (2022).

[24] Wang, Z. W. et al. Inhibition mechanism of mining-induced fracture development in Thick coal seam with super-thick alluvium and thin bedrock. *Coal Sci. Technol. Magazine*. **43**, 67–70 (2022).

[25] Jia, B., Chen, H., Li, Z. & Tang, Z. Overlying rock activity laws and influential ranges of coal seam mining in stubble areas. *Geofluids* **2022**, 3399984 (2022).

[26] Wang, Y. L. et al. Failure mechanism and movement characteristics of overlying strata in Longwall mining face with Thick aquifer. *Rock Mech. Rock Eng.* **57**, 6787–6809 (2024).

[27] Zhang, C. S. et al. Application research of digital close-range photogrammetry in similarity simulation experiment. *Coal Sci. Technol.* **42**, 93–96 (2014).

[28] Huang, Y. S. Movement and deformation laws of overlying strata and surface under cut-off and backfill mining in shallow coal seam with thin bedrock. *China Univ. Min. Technology.* (2023).

[29] Lu, Z. J. Development height of water-flowing fractured zone in overlying strata of coal seam with thin bedrock and Thick loose layer. *Min. Saf. Environ. Prot.* **50**, 105–110 (2023).

[30] Ju, J. F. et al. Key stratum movement monitoring and five-stage of rock movement: a case study of Hongqinghe coal mine. *J. China Coal Soc.* **47**, 611–622 (2022).

[31] Christianson, M., Board, M. & Rigby, D. UDEC simulation of triaxial testing of lithophysal tuff. Golden Rocks The 41st US Symposium on Rock Mechanics (USRMS). *American Rock Mechanics Association, Alexandria.* (2006).

[32] Fabjan, T., Ivars, D. M. & Vukadin, V. Numerical simulation of intact rock behaviour via the continuum and Voronoi tessellation models: a sensitivity analysis. *Acta Geotech. Slov.* **12**, 4–23 (2015).

